# The morphology of the immature stages of two rare *Lixus* species (Coleoptera, Curculionidae, Lixinae) and notes on their biology

**DOI:** 10.3897/zookeys.604.9018

**Published:** 2016-07-11

**Authors:** Filip Trnka, Robert Stejskal, Jiří Skuhrovec

**Affiliations:** 1 Department of Ecology and Environmental Sciences, Faculty of Science, Palacký University Olomouc, Šlechtitelů 27, CZ-783 71 Olomouc, Czech Republic; 2 Administration of Podyji National Park, Na Vyhlídce 5, CZ-669 02 Znojmo, Czech Republic; 3 Group Function of Invertebrate and Plant Biodiversity in Agrosystems, Crop Research Institute, Drnovská 507, CZ-161 06 Praha 6 – Ruzyně, Czech Republic

**Keywords:** Weevil, mature larva, pupa, larval development, life cycle, host plant, Cichorium intybus, Rumex thyrsiflorus, Central Europe, Palaearctic region

## Abstract

The mature larvae and pupae of Lixus (Ortholixus) bituberculatus Smreczyński, 1968 and Lixus (Dilixellus) neglectus Fremuth, 1983 (Curculionidae: Lixinae: Lixini) are described and compared with known larvae of 21 other *Lixus* and 2 *Hypolixus* taxa. The mature larva and pupa of *Lixus bituberculatus* are the first immature stages described representing the subgenus *Ortholixus*. The larva of *Lixus neglectus*, in the subgenus *Dilixellus*, is distinguished from the known larvae of four species in this subgenus by having more pigmented sclerites on the larval body. All descriptions of mature larvae from the tribe Lixini, as do all known species from the tribe Cleonini, fit the diagnosis of the mature larva of the Lixinae subfamily. Furthermore, new biological information of these species in the Czech Republic, Slovakia and Romania is provided. For *Lixus bituberculatus*, a chicory, *Cichorium intybus* L. (Asteraceae), is identified as a host plant, and *Lixus neglectus* is found on dock *Rumex thyrsiflorus* Fingerh. (Polygonaceae). Both species are probably monophagous or oligophagous. Adults of *Lixus bituberculatus* often inhabit host plants growing in active, dry and sunny pastures with sparse patches without vegetation, being mostly active during the night in April/May and then again in September, when the highest activity levels are observed. Adults of *Lixus neglectus* inhabit dry grasslands on sandy soils with host plants, being active during the day from May to September, with the highest level of activity in May/June and September. The larvae of both species are borers in the stem and root of the host plant, and they pupate in root or root neck. Adults leave the pupation cells at the end of summer and do not hibernate in the host plants. Finally, Romania is a new geographic record for *Lixus bituberculatus*.

## Introduction

The genus *Lixus* Fabricius, 1801, belongs to the tribe Lixini Schoenherr, 1823 in the subfamily Lixinae Schoenherr, 1823 (family Curculionidae Latreille, 1802), and approximately 500 species have been described globally (Ter-Minasian 1967). Approximately 170 *Lixus* species in 12 subgenera are found in the Palaearctic region; only *Eutulomatus* Desbrochers des Loges, 1893 and *Parileomus* Voss, 1939 do not occur in Europe (Gültekin and Fremuth 2013). The biology of these species is partially known but has primarily only been studied recently (e.g., Nikulina 1989; Volovnik 1994, 2007; Gültekin 2007; Gosik and Wanat 2014; Skuhrovec and Volovnik 2015). The immature stages of *Lixus* species usually develop in the stems (Meregalli 2014) or the roots of plants (Dieckmann 1983) but sporadically develop in the seed capsule (Gültekin 2005) or petiole (Gültekin 2007). Some species from this genus are considered to be agricultural pests (e.g., *Lixus incanescens* Boheman, 1835 in Manole 1990; others in Volovnik 1988; Nikulina 1989), but others could be used for the biological control of selected weeds (e.g., *Lixus filiformis* (Fabricius, 1781) for musk thistle; Gültekin 2004) or have already been used for these purposes (e.g., *Lixus cardui* Olivier, 1807 in Australia; Nikulina and Gültekin 2011). Detailed morphological descriptions have been published for the larvae of 21 *Lixus* species, with detailed descriptions of the pupae being available for only eight species (see Scherf 1964; Lee and Morimoto 1988; May 1994; Nikulina 2001, 2007; Zotov 2009a, b; Nikulina and Gültekin 2011; Gosik and Wanat 2014; Skuhrovec and Volovnik 2015).

*Lixus bituberculatus* Smreczyński, 1968 belongs to the subgenus *Ortholixus* Reitter, 1916, which includes 18 species in the Palaearctic region (Gültekin and Fremuth 2013). This species is among the rarest of the genus *Lixus*, only distributed in Hungary, Slovakia and Bulgaria (Gültekin and Fremuth 2013; Stejskal and Trnka 2014). The biology and host plant of *Lixus bituberculatus* were completely unknown, and its immature stages have never been described.

*Lixus neglectus* Fremuth, 1983 belongs to the subgenus *Dilixellus* Reitter, 1916, which includes 31 species in the Palaearctic region (Gültekin and Fremuth 2013), and the distribution of this central European endemic weevil includes a relatively small area (ca. 5000 km^2^) in Austria, the Czech Republic and Slovakia at the confluence of the Dyje and Morava Rivers (Gültekin and Fremuth 2013; Trnka and Stejskal 2014). The biology and plant associations of *Lixus neglectus* are better known than previously described species, and several authors reported the occurrence of adults on garden sorrel (*Rumex acetosa* L.) (e.g., Fremuth 1983; Koch 1992; Böhme 2001; Trnka and Stejskal 2014). The immature stages of *Lixus neglectus* have never been described.

Knowledge of the immature stages and life histories of both species is important for taxonomy as well as practical applications and can help to more effectively protect these species. In this paper, we describe the immature stages of both species and provide details of their life history based on field observations in the Czech Republic, Slovakia and Romania.

## Materials and methods

The material used to describe the immature stages was collected, and field observations were conducted in the localities mentioned below:

**Lixus (Ortholixus) bituberculatus Smreczyński, 1968**

**Material examined. ROMANIA**: **Caraș-Severin County**: Sfânta Elena env.; 44°40'24.1"N, 21°43'2.0"E; survey dates: 9-VI-2012, 1–2-IX-2012, 18–23-V-2013, 5-VIII-2014 (3 larvae), 6-VIII-2014 (2 larvae, 1 pupa), 8-VIII-2014 (7 larvae, 3 pupae); all leg. & det. F. Trnka, coll. J. Skuhrovec. Habitats: pastures (cattle, sheep, goats), road margins and dry grasslands. Bedrock: limestone. Altitude: 400 m a. s. l. (see Fig. 21). **SLOVAKIA**: **Rimavská Sobota District**: Gemerské Dechtáre env.; 48°15'22.03"N, 20°2'12.44"E; 11-IV-2015. Habitats: pastures (cattle and sheep), road margins and dry grasslands. Bedrock: quaternary eolithic sediments (sand and loess). Altitude: 206 m a. s. l.

**Lixus (Dilixellus) neglectus Fremuth, 1983**

**Material examined. CZECH REPUBLIC**: **Břeclav District**: Lanžhot env.; 48°41'21.04"N, 16°56'3.40"E; survey date: 19-VII-2014. Habitat: dry grassland and blown sand, THG01 *Potentillo heptaphyllae*-*Festucetum rupicolae* (Chytrý et al. 2010). Bedrock: quaternary alluvial sediments (sand). Altitude: 164 m a. s. l. (see Fig. 41); Kostice env.; 48°45'54.60"N, 16°56'36.54"E; survey date: 9-VI-2015. Habitat: grassy road embankment. Bedrock: artificial structure (sandy gravel). Altitude: 168 m a. s. l. **SLOVAKIA**: **Trnava District**: Sekule env.; 48°37'8.60"N, 16°59'21.03"E; survey dates: 13-VII-2014 (5 mature larvae, 2 younger larvae, 1 pupa) and 25-VII-2014 (2 pupae reared from larvae collected on 13-VII-2014); all leg. R. Stejskal & F. Trnka, det. R. Stejskal & F. Trnka, coll. J. Skuhrovec. Habitat: dry meadow. Bedrock: quaternary alluvial sediments (sand). Altitude: 158 m a. s. l.

Rearing and life cycle observations were conducted during the 2014–2015 vegetation growing seasons. Laboratory observations were conducted in Olomouc (49°35'36"N, 17°15'3"E) and in Znojmo, Czech Republic (48°51'31"N, 16°2'40"E).

Part of the larval and pupal material was preserved in Pampel fixation liquid (4 parts glacial acetic acid, 6 parts 4% formaldehyde, 15 parts 95% ethyl alcohol and 30 parts distilled water) and used for the morphological descriptions. These specimens are now deposited in the Group Function of Invertebrate and Plant Biodiversity in Agrosystems of the Crop Research Institute (Prague, Czech Republic). Plants were identified by the collectors. Slides were prepared following May (1994) as follows: a larva was decapitated, its head was cleared in a 10% potassium hydroxide (KOH) solution and then rinsed in distilled water. After clearing, the mouth parts were separated from the head capsule. The head capsule and the mouth parts were mounted on permanent microscope slides in Euparal. The body parts (thorax and abdomen) were mounted on temporary microscope slides in 10% glycerine.

The observations and measurements were made using a light microscope with calibrated oculars (Olympus BX 40 and Nikon Eclipse 80i), and the following characteristics were measured for each larva: head width, length of the body (larvae fixed in a C-shape were measured in segments), width of the widest part of the body (metathorax or abdominal segments I–IV).The length and width of the widest part of the body was measured for each pupa. The thorax and abdomen were not sclerotised, and it is unlikely that the fixation process altered the weevils’ proportions; measurements of these parts are given for comparison purposes only.

Drawings were made with a drawing tube on a light microscope and processed by a computer program (Adobe Photoshop, Corel Photo-Paint 11, GIMP 2). The thoracic spiracle is located on the prothorax near the boundary of the prothorax and mesothorax, as shown in the drawing (see Figs 8, 29), but it is of mesothoracic origin (Marvaldi et al. 2002, Marvaldi 2003). The drawings show the thoracic and abdominal spiracles (see Figs 8–10, 29–31). The numbers of setae are given for one side of the bilateral structures.

We used the terms and abbreviations for the setae of the mature larva and pupa studied in Scherf (1964), May (1977, 1994) and Marvaldi (1998a, 1999).

The count of some of the setae on the epipharynx (especially *ams* and *mes*) have not been completely resolved. According to Marvaldi (1998a, 1999), the standard status of the epipharynx in weevils is 2 *ams* and 3 *mes*, but when the position of the distal *mes* is very close to the anterior margin, they appear as *ams*. The decision was finally made to add this problematic seta to the latter group (*ams*), and the position of this seta is similar to that in other genera, e.g., in *Coniocleonus* Motschulsky or *Tychius* Germar. We did not follow Stejskal et al. (2014) and Skuhrovec et al. (2014), who accepted the standard status in weevils and counted the seta as *mes*, but we followed Trnka et al. (2015) and Skuhrovec et al. (2015), e.g., in *Adosomus* Faust or *Sibinia* Germar.

## Results

### Lixus (Ortholixus) bituberculatus

Taxon classificationAnimaliaColeopteraCurculionidae

Smreczyński, 1968

#### Description of mature larva.

*Measurements* (in mm). Body length: 6.5–10.4 (mean 9.2). The widest part of the body (metathorax and abdominal segments I–II) measuring up to 2.8. Head width: 1.4–1.7 (mean 1.5).

*General*. Body stocky, slightly curved, rounded in cross section (Fig. 7). Cuticle finely spiculate.

*Colouration*. Head light brown or brown with a distinct pale pattern around the frontal suture (Figs 7, 19). All thoracic and abdominal segments are white with a light brown, elongate stripe on the dorsum of the pronotum (Fig. 7).

*Vestiture*. Setae on body thin, relatively short, light yellow or orange.

**Figure 1.**
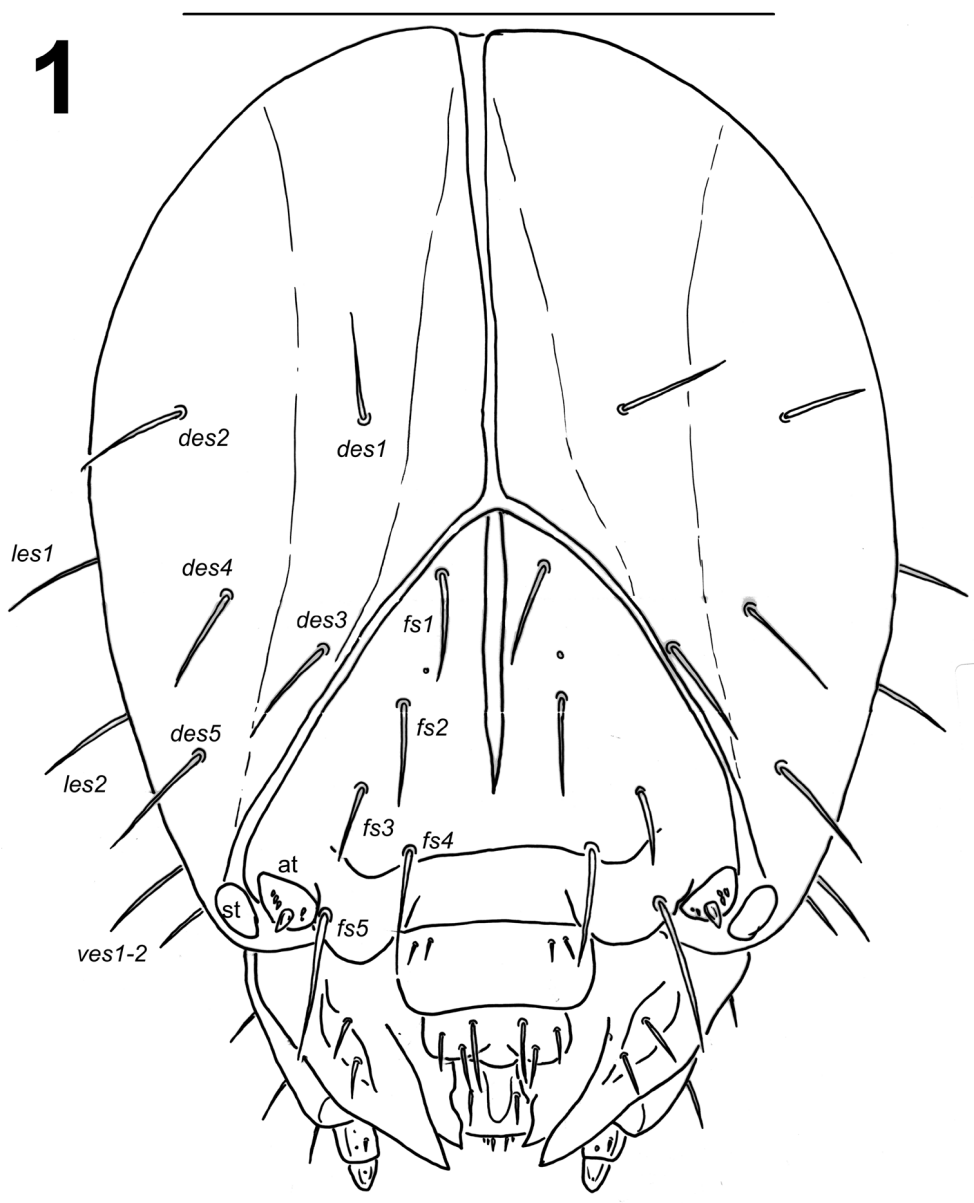
*Lixus bituberculatus* mature larva head, dorsal view. Scale bar: 1 mm.

*Head capsule* (Fig. 1). Head suboval, flattened laterally, endocarinal line long more than half length of frons. Frontal sutures distinct, extended to the antennae. Single stemma (st) in the form of a slightly pigmented spot located anterolaterally on each side. *Des1* and *des2* located in the upper part of the central part of the epicranium, *des1* near the middle part of epicranium, and *des2* near the side of the epicranium, *des3* located anteriorly near the frontal suture, *des4* located in the central part of the epicranium, *des5* located anterolaterally; all *des* long, subequal in length (Fig. 1). *Fs1* and *fs2* placed medially, *fs3* located anteromedially, *fs4* located anterolaterally, and *fs5* located laterally, close to the epistoma; all setae relatively long, *fs4* slightly longer than *fs1-3* and *fs5* distinctly longer than *fs4* (Fig. 1). *Les1–2* as long as *des1*; *ves1–2* as long as *fs3*. Epicranial area with sensilla undistinct.

Antennae located at the end of the frontal suture on each side, membranous and slightly convex basal article bearing one conical triangular sensorium, relatively long; basal membranous article with 5 sensilla different in both shape and length (Fig. 4).

**Figures 2–3.**
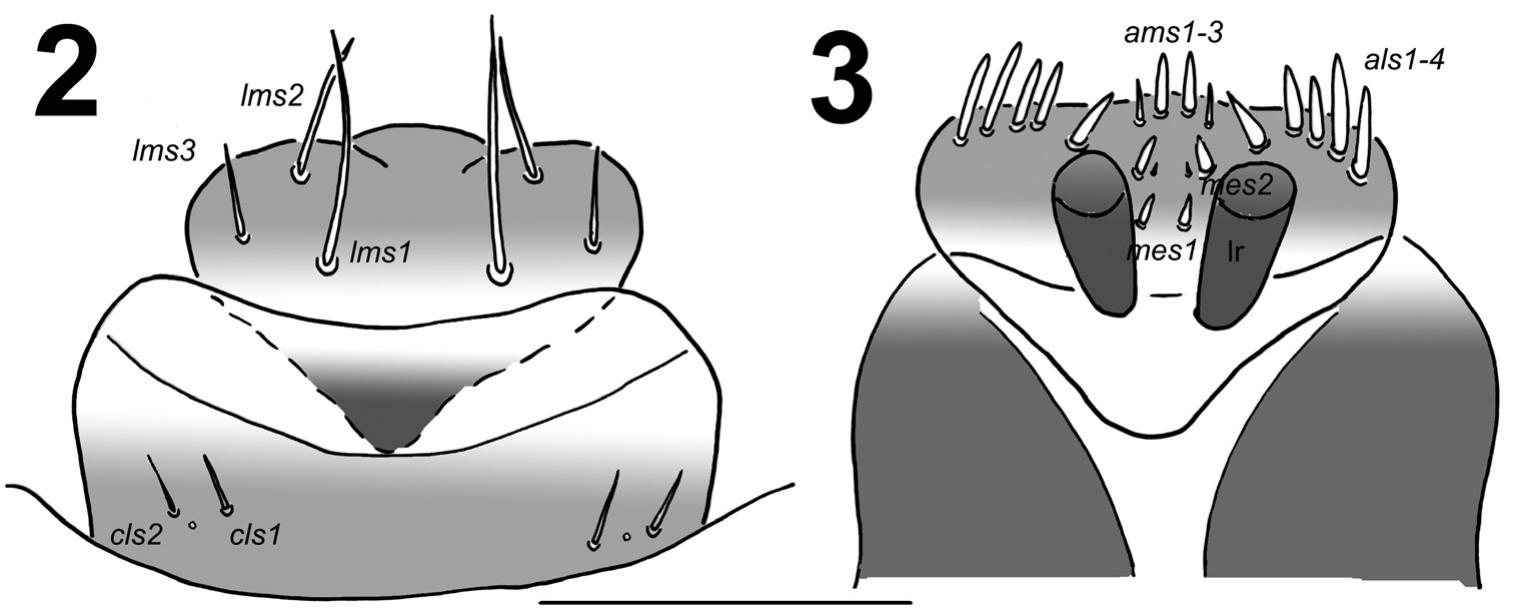
*Lixus bituberculatus* mature larva. **2** Labrum and clypeus **3** Epipharynx. Scale bar: 0.5 mm.

*Clypeus* (Fig. 2) approximately 2.1 times as wide as long with 2 relatively long *cls*, almost equal in length, localized posterolaterally and 1 sensillum; anterior margin rounded to the inside.

**Figures 4–5.**
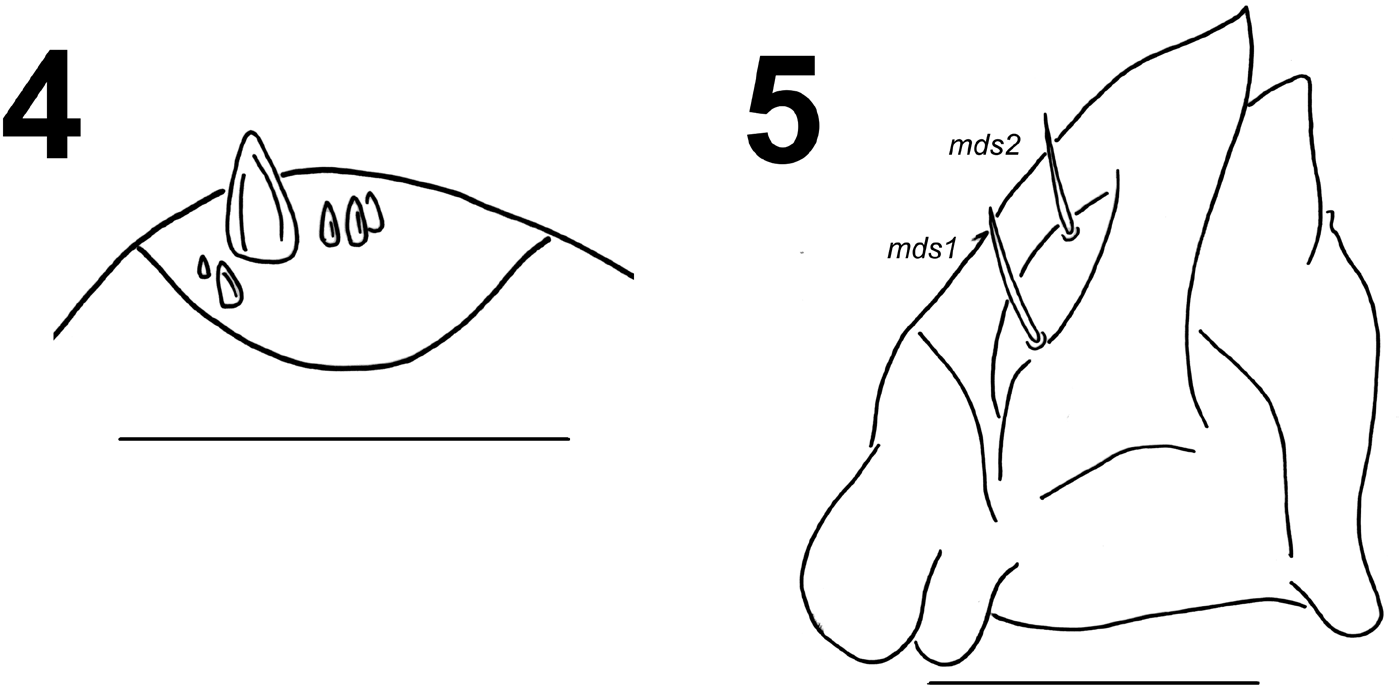
*Lixus bituberculatus* mature larva head. **4** Antenna **5** Right mandible. Scale bars: 0.1 mm (**4**) and 0.2 mm (**5**).

**Figure 6.**
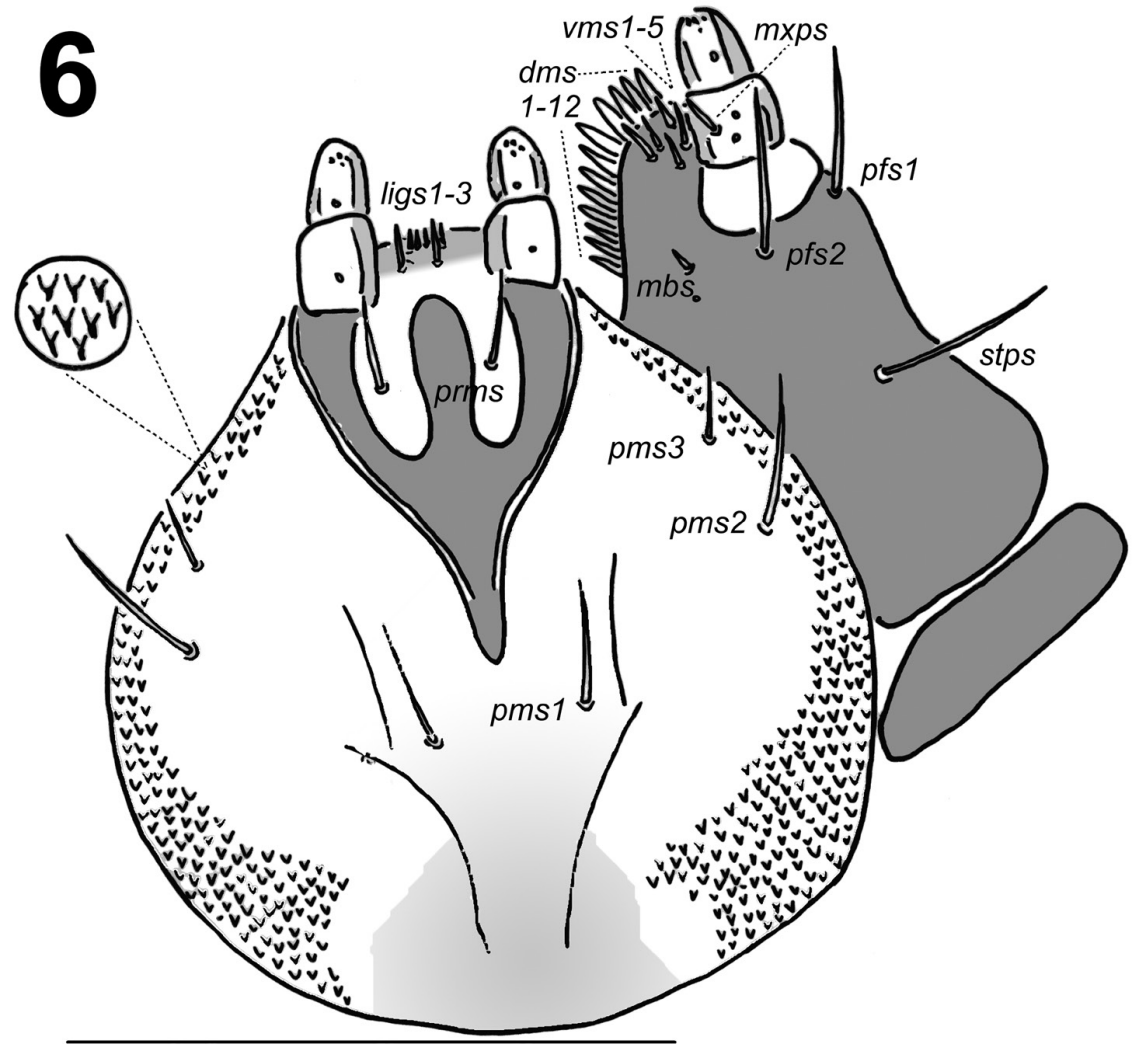
*Lixus bituberculatus* mature larva head, maxillo-labial complex, ventral view. Scale bar: 0.5 mm.

*Mouth parts*. Labrum (Fig. 2) approximately 2 times as wide as long, with 3 pairs of piliform *lms*, of different lengths; *lms3* distinctly shorter than very long *lms1* and long *lms2*; *lms1* located close to the margin with clypeus, *lms2* located anteromedially and *lms3* located anterolaterally; anterior margin double sinuate. Epipharynx (Fig. 3) with 4 pairs of blunt, finger-like *als*, unequal in length, *als1–2* distinctly shorter than *als3–4*; 3 pairs of *ams*, *ams1* and *ams2* distinctly shorter than *ams3*, *ams1* and *ams2* piliform, and *ams3* blunt, finger-like; 2 pairs of short, blunt *mes* and one sensilla close to *mes2*, located close to lr; labral rods (lr) elongated, converging anteriorly. Mandibles (Fig. 5) relatively broad, bifid, teeth of unequal height; slightly truncate; both *mds* relatively long, piliform. Maxilla (Fig. 6) stipes with 1 *stps*, 2 *pfs* and 1 *mbs*; very long *stps* distinctly longer than long *pfs1–2*, *mbs* very short; mala with 12 bacilliform *dms* of two different lengths (6 very long and 6 relatively long); 5 short *vms*, almost equal in length; *vms* distinctly shorter than *dms*. Maxillary palpi with two palpomeres; basal palpomere with 1 very short *mxps* and two sensilla; length ratio of basal and distal palpomeres: 1:0.7; distal palpomere with one sensillum and a group of conical, apical sensorial papillae. Praelabium (Fig. 6) heart-shaped and distinctly elongated, with 1 relatively long *prms*; ligula with sinuate margin and 3 piliform micro *ligs*, unequal in length; premental sclerite well visible. Labial palpi with two palpomeres; length ratio of basal and distal palpomeres: 1:0.7; distal palpomere with one sensillum and short, apical sensorial papillae; basal palpomere with 1 ventral sensillum. Postlabium (Fig. 6) with 3 *pms*, *pms1* located anteriorly, remaining two pairs laterally; relatively long, almost of equal length, *pms3* distinctly shorter than *pms1* and *pms2*; surface of postlabium densely covered by distinct asperities.

*Thorax*. Prothorax distinctly smaller than meso- and metathorax. Metathorax almost of equal length as abdominal segments I–IV. Spiracle bicameral. Prothorax (Fig. 8) with 10 *prns* unequal in length, 8 of them on weakly pigmented premental sclerite, which is subdivided medially into two triangular plates, next two *prns* placed below; 2 *ps* and 2 *eus*. Mesothorax (Fig. 8) with 1 *prs*; 4 *pds* unequal in length, *pds2* distinctly shorter than the remaining three setae; 1 short *as*; 2 short to very short *ss*; 1 *eps*; 1 *ps* and 2 *eus*. Chaetotaxy of metathorax (Fig. 8) identical to that of mesothorax. Each pedal area of the thoracic segments well separated and pigmented, with 7 long *pda*, 6 of which on pigmented area, unequal in length.

**Figure 7.**
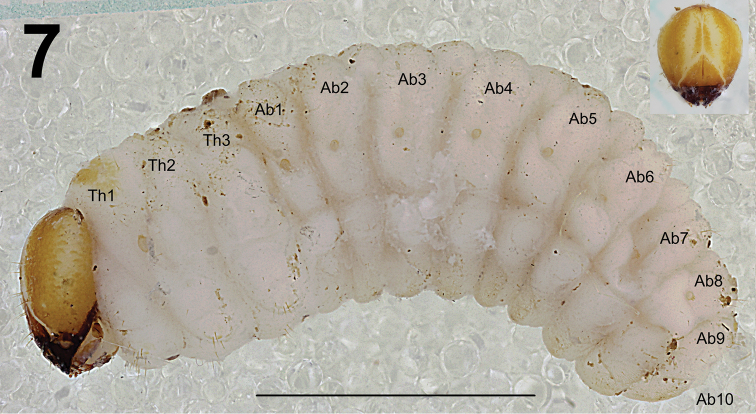
*Lixus bituberculatus* mature larva habitus, lateral view. Scale bar: 3 mm.

**Figures 8–10.**
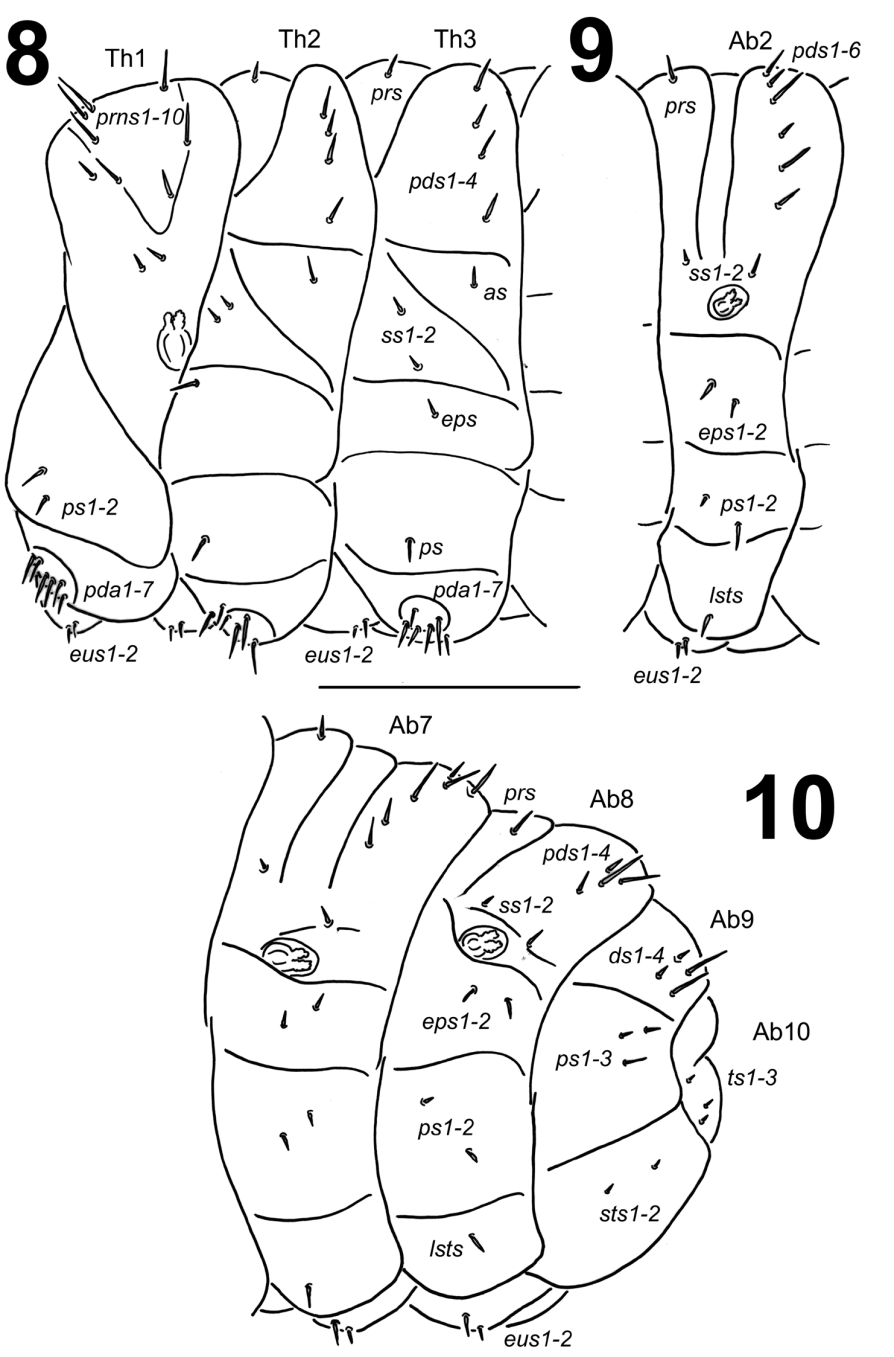
*Lixus bituberculatus* mature larva habitus. **8** Lateral view of thoracic segments **9** Lateral view of abdominal segment II. **10** Lateral view of abdominal segments VII–X. Scale bar: 1 mm.

*Abdomen*. Abdominal segments I–IV of almost equal length, subsequent abdominal segments decreasing gradually to the terminal parts of the body. Abdominal segment X reduced to four anal lobes of unequal size, the dorsal being distinctly the largest, the lateral pair equal in size, and the ventral lobe very small. Anus located terminally. Spiracles bicameral, the eight abdominal spiracles located laterally, close to the anterior margin of abdominal segments I–VIII. Abdominal segments I–VII (Figs 9–10) with 1 *prs*; 6 *pds*, *pds3* and *pds5* the longest one; 2 *ss* of unequal length, *ss1* very short, *ss2* as long as *pds6*; 2 *eps* of almost equal length; 2 relatively short *ps* of unequal length, *ps1* very short to minute, *ps2* short; 1 *lsts* and 2 *eus*. Abdominal segment VIII (Fig. 10) with 1 *prs*; 4 *pds*, *pds1* and *pds6* lacking, *pds3* and *pds5* less than half of length of the two remaining setae; 2 *ss* of unequal length, *ss1* very short, *ss2* long as *pds6*; 2 *eps* of almost equal length; 2 short *ps* of equal length; 1 *lsts* and 2 *eus*. Abdominal segment IX (Fig. 10) with 4 *ds* (*ds1* and *ds3* very short, *ds 2* and *ds4* long); 2 short and 1 very short *ps* and 2 very short to micro *sts*. Abdominal segment X (Fig. 10) with 2 microsetae and 1 seta (*ts*) on each lateral anal lobe.

#### Description of pupa.

*Measurements* (in mm). Body length: 8.0–10.4 (♂ 8.0–10.4; ♀ 9.8). The widest part of the body, commonly between the apex of the meso- or metafemora: 2.6–3.5.

*Colouration*. Body white to yellowish (Fig. 20).

**Figures 11–13.**
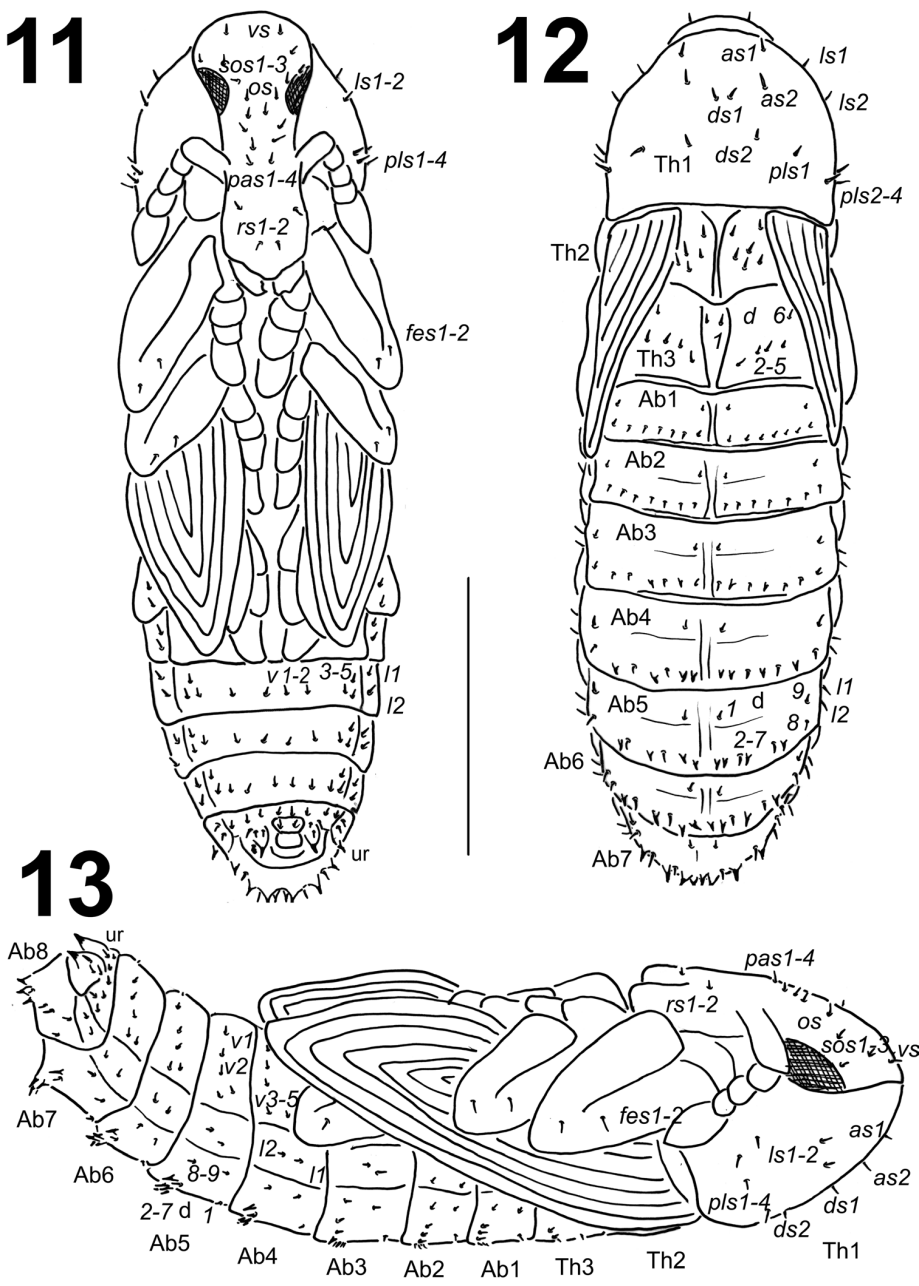
*Lixus bituberculatus* pupa habitus. **11** Ventral view **12** Dorsal view **13** Lateral view. Scale bar: 3 mm.

*Morphology* (Figs 11–13, 20). Body stocky, elongated, white or yellowish. Cuticle smooth. Rostrum relatively long, approximately 2.7 to 3.0 times as long as wide, extending beyond the mesocoxae; females with slightly thinner rostrum than males. Antennae relatively long and stout. Pronotum from 1.2 to 1.3 times as wide as long. Mesonotum and metanotum of almost equal length. Abdominal segments I–III of almost equal length; abdominal segment VI semicircular and subsequent abdominal segments diminish gradually to the end of the body. Abdominal segments VII–IX distinctly smaller than other abdominal segments. Gonotheca (abdominal segment IX) in females (1 specimen) bilobed.

**Figures 14–21.**
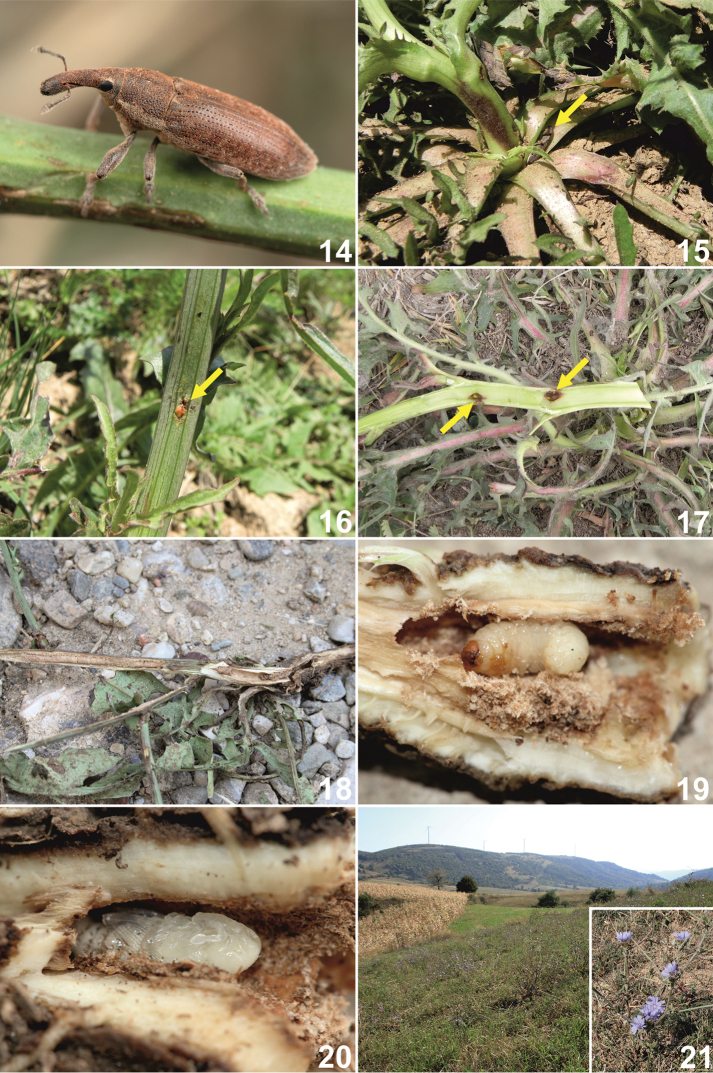
Habitats, adults, immature stages and life cycle of *Lixus bituberculatus*. **14** Adult **15** Adult hiding in host plant rosette **16** Ovipositional mark **17** Eggs in the host plant stem **18** Feeding marks in the stem **19** Mature larva in the root crown **20** Pupa and pupation cell **21** Habitat in Romania and *Cichorium intybus* host plant.

*Chaetotaxy* (Figs 11–13). Setae relatively short, unequal in length, light yellow or orange, some setae on abdominal segments III–VIII distinctly stronger and located on protuberances. Setae well visible. Head capsule includes 1 *vs*, 3 *sos*, 1 *os* and 4 *pas*. Rostrum with 2 *rs*, *rs1* located below antenna, *rs2* placed on the anterior margin. Setae on head capsule and rostrum straight, both *rs* and all *pas* distinctly shorter than the remaining setae on head, thoracic and abdominal segments. Pronotum with 2 *as*, 2 *ds*, 2 *ls* and 4 *pls*. Dorsal parts of mesothorax with 1 seta located posteromedially, 1 seta located posterolaterally and 4 setae located along its anterior margin. Chaetotaxy of metathorax identical to that of mesothorax. Each femoral apex with 2 *fes*. Dorsal parts of abdominal segments I–VIII each with 2 pairs of setae located posteriorly (*d1*, *d9*) and 7 pairs (*d2–8*) located along their anterior margins. Setae *d2–3*, *d5* and *d7* (on abdominal segments IV–VIII) short, thorn-like, located on protuberances; on abdominal segment III only setae *d3* and *d5*. Remaining setae short to very short, hair-like; all setae very short on abdominal segments I–II. Abdominal segments I–VII with groups of 2 lateral setae and 5 pairs of ventral setae. Dorsal part of abdominal segment VIII with 1 seta located posteriorly (*d9*) and 7 pairs (*d2–8*) located along its anterior margin; *d3*, *d5* and *d7* thorn-like, located on protuberances; remaining setae elongated. Abdominal segment VIII with groups of 2 lateral setae and 5 short ventral setae. Abdominal segment IX with 2 pairs of ventral microsetae and 1 pair of short, thin setae. Urogomphi elongated, triangular.

#### Biology and ecology.

*Habitats*. Adults (Fig. 14) prefer dry and sunny habitats such as dry grasslands, meadows often with grazing or mowing (Fig. 21), and road margins with specific disturbance regimes (trampling by movement of cattle or vehicles, etc.).

*Adult behaviour*. During the day, adults stay among the rosette leaves of the host plant (Fig. 15) near the stem base. Adults were usually observed by sweeping the host plants at night. Data were collected from April to September with the exception of July. The maximum number of records occurred in late summer.

*Host plants*. Adults and larvae were observed feeding on chicory *Cichorium intybus* L. (Asteraceae), in the studied localities (Fig. 21). Nevertheless, J. Krátký and J. Pelikán (in litt.) also found adult *Lixus bituberculatus* on *Crepis* sp. and *Picris* sp. during night sweeping and a pupa in the root of *Picris* sp. in Slovakia (Hajnáčka env. and Bajtava env.).

*Life cycle. Lixus bituberculatus* is an univoltine species. Adults feed on leaves, but larval development occurs in the basal part of the stem and in the root (Figs 18–19). Females of *Lixus bituberculatus* bite the lower part of the stem of the host plant near the ground and lay one egg in the hole (Figs 16–17). Usually, only one larva was found to occupy a plant, but rarely, there were two (one in the stem and another in the root crown). Mature larvae were found from July to August. Pupation occurs in the root neck or root (Fig. 20), and fresh adults can be found (inside plants) from middle of August. The exit hole is situated in the upper part of the pupation cell. Adults do not hibernate in the host plants. Most likely, hibernation occurs in the leaf litter, among dry plant debris or in the topsoil.

*Rearing of the larvae*. For laboratory breeding, 10 mature larvae were collected on August 8^th^, 2014, but only three pupated under our laboratory conditions. The remaining seven larvae primarily died due to drying of the host plants. The first fresh adult hatched on September 12^th^ and the other two on September 15^th^, 2014.

### Lixus (Dilixellus) neglectus

Taxon classificationAnimaliaColeopteraCurculionidae

Fremuth, 1983

#### Description of mature larva.

*Measurements* (in mm). Body length: 10.5–13.5 (mean 12.5). The widest part of the body (metathorax and abdominal segments I–II) measuring up to 3.3. Head width: 1.8–2.1 (mean 2.0).

*General*. Body stocky, slightly curved, rounded in cross section (Fig. 28), densely covered by distinct asperities (mainly dorsal and ventral parts). Cuticle finely spiculate.

*Colouration*. Head light brown or brown (Figs 28, 38). All thoracic and abdominal segments are white with a light brown elongate stripe on the dorsum of the pronotum (Fig. 28).

*Vestiture*. Setae on body thin, relatively long to very long, light yellow or orange.

**Figure 22.**
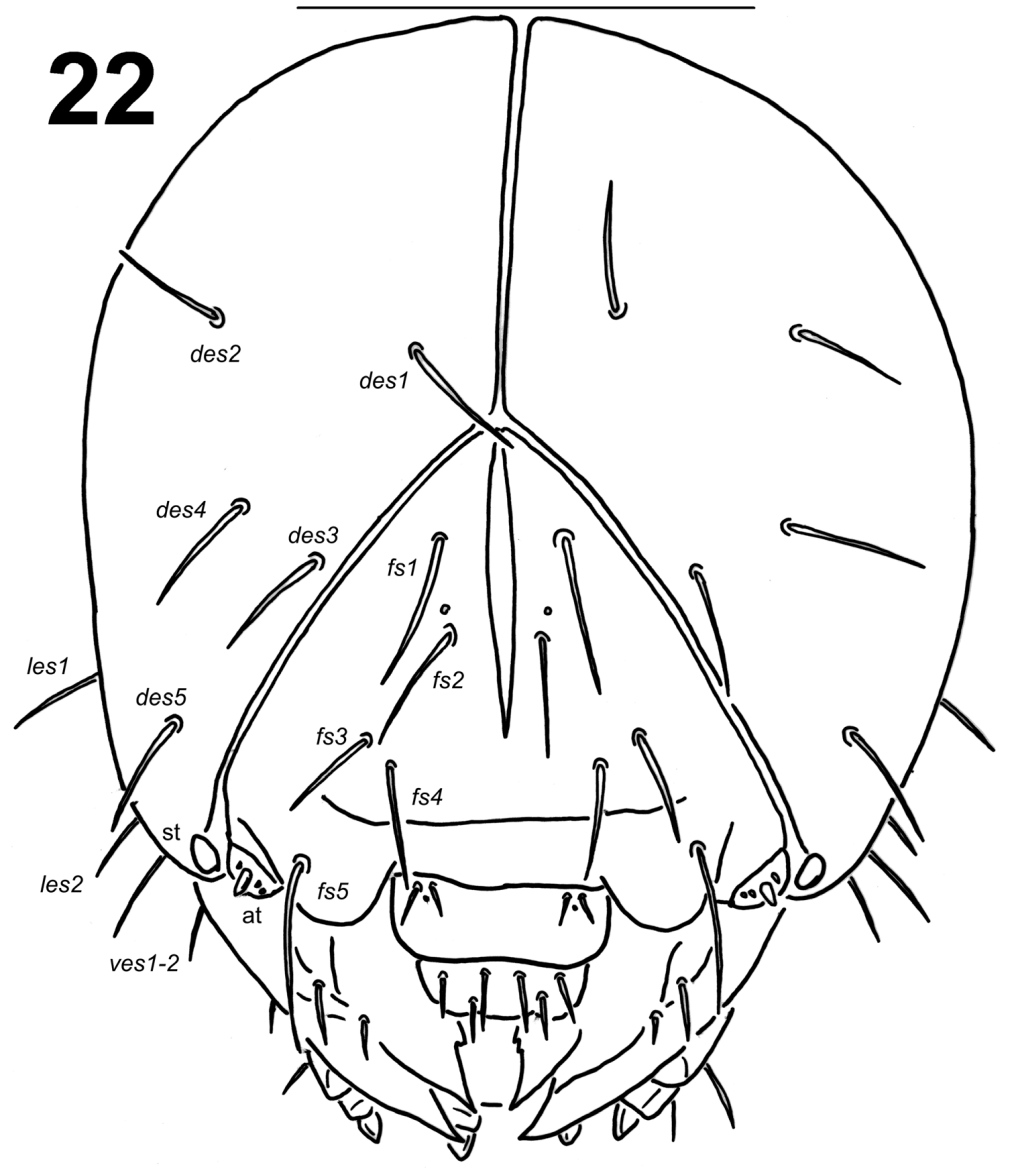
*Lixus neglectus* mature larva head, dorsal view. Scale bar: 1 mm.

*Head capsule* (Fig. 22). Head suboval, flattened laterally, endocarinal line long more than half length of frons. Frontal sutures distinct, extended to the antennae. Single stemma (st) in the form of a slightly pigmented spot, located anterolaterally on each side. *Des1* and *des2* located in the upper part of the central part of the epicranium, *des1* near the middle part of the epicranium, and *des2* near the side of the epicranium, *des3* located anteriorly near the frontal suture, *des4* located in the central part of the epicranium, *des5* located anterolaterally; all *des* long, subequal in length (Fig. 22). *Fs1* and *fs2* placed medially, *fs3* located anteromedially, *fs4* located anterolaterally, and *fs5* located laterally, close to the epistoma; all setae long to extremely long, *fs1* extremely long, *fs4* and *fs5* very long, distinctly longer than *fs2* and *fs3*, but shorter than *fs1* (Fig. 22). *Les1–2* as long as *des1*; *ves1–2* as long as *fs3*. Epicranial area with sensilla undistinct.

*Antennae* located at the end of the frontal suture on each side, membranous and slightly convex basal article bearing one conical triangular sensorium, relatively long; basal membranous article with 3 sensilla different in both shape and length (Fig. 25).

**Figures 23–24.**
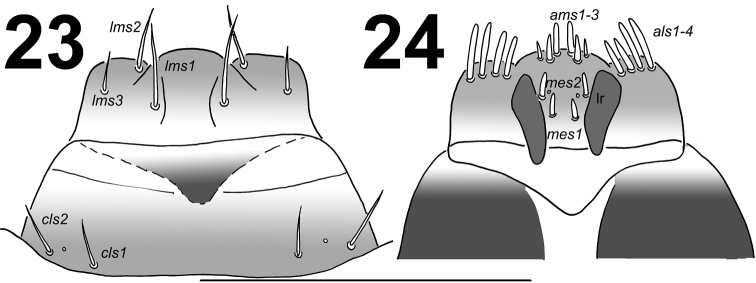
*Lixus neglectus*mature larva. **23** Labrum and clypeus **24** Epipharynx. Scale bar: 0.5 mm.

*Clypeus* (Fig. 23) approximately 2.5 times as wide as long with 2 long *cls*, almost equal in length, localized posterolaterally and 1 sensillum; anterior margin rounded to the inside; median part covered by thorn-shaped asperities.

**Figures 25–26.**
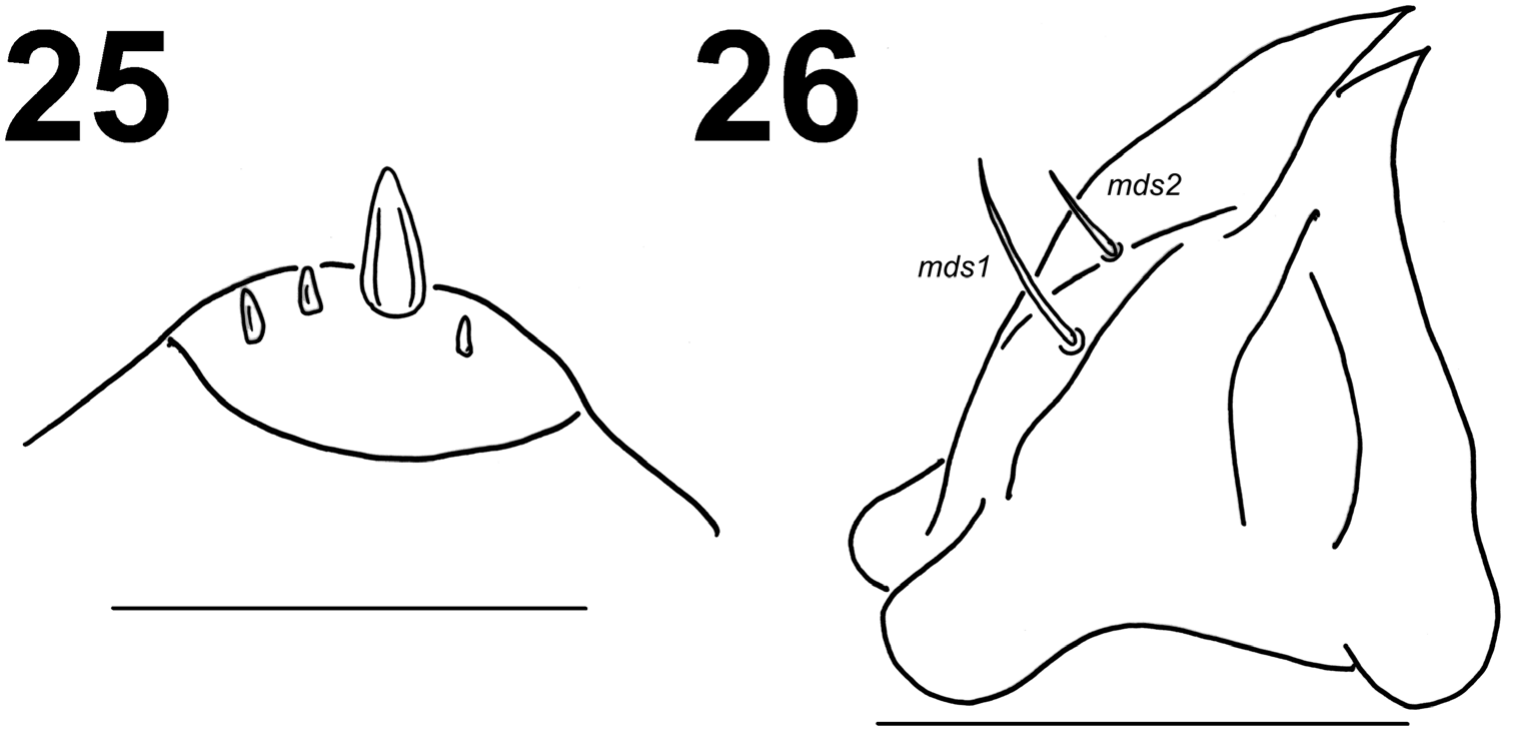
*Lixus neglectus* mature larva head. **25** Antenna **26** Right mandible. Scale bars: 0.1 mm (**25**) and 0.5 mm (**26**).

**Figure 27.**
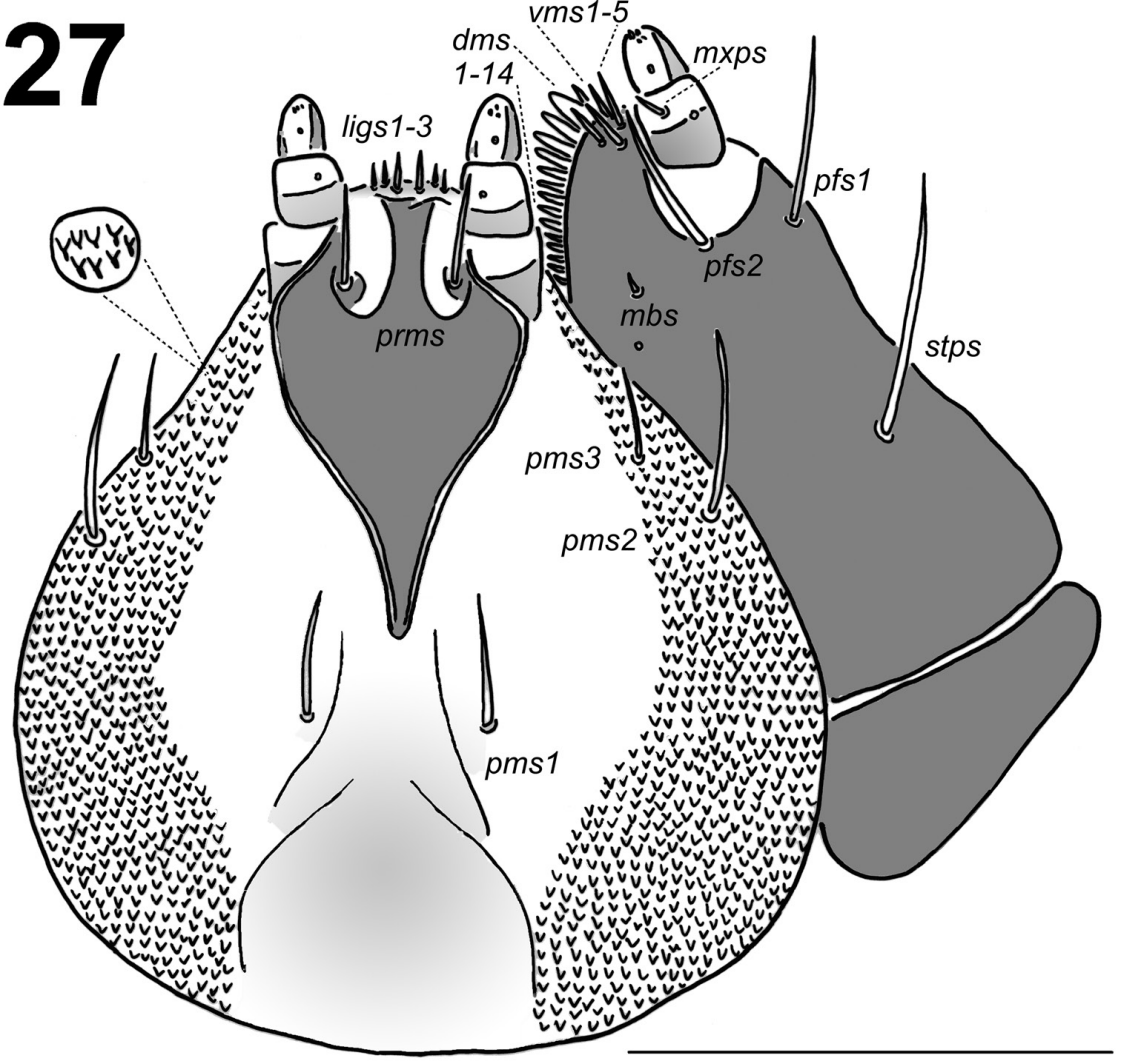
*Lixus neglectus* mature larva head, maxillo-labial complex, ventral view. Scale bar: 0.5 mm.

*Mouth parts*. Labrum (Fig. 23) approximately 3 times as wide as long, with 3 pairs of piliform *lms*, of different lengths; *lms3* distinctly shorter than very long *lms1* and long *lms2*; *lms1* placed close to the margin with clypeus, *lms2* located anteromedially and *lms3* located anterolaterally; anterior margin double sinuate. Epipharynx (Fig. 24) with 4 pairs of blunt, finger-like *als*, unequal in length, *als1–2* distinctly shorter than *als3–4*; 3 pairs of *ams*, *ams1* and *ams3* distinctly shorter than *ams2*, *ams1* and *ams3* piliform, and *ams2* blunt, finger-like; 2 pairs of short, blunt *mes* and one sensilla close to *mes2*; labral rods (lr) elongated, converging anteriorly, distinctly pigmented. Mandibles (Fig. 26) relatively broad, bifid, teeth of unequal height; slightly truncate; *mds1* very long, *mds2* distinctly short, piliform. Maxilla (Fig. 27) stipes with 1 *stps*, 2 *pfs* and 1 *mbs*; *stps* and *pfs1–2* very long, almost equal in length, *mbs* very short; mala with 14 bacilliform *dms* in two different lengths (1–4 long, blunt and 5–14 very long and blunt, with a tendency to be longer and less blunt); 5 relatively long *vms*, almost equal in length; *vms* distinctly shorter than *dms*. Maxillary palpi with two palpomeres; basal palpomere with 1 very short *mxps* and two sensilla; length ratio of basal and distal palpomeres: 1:0.8; distal palpomere with one sensillum and a group of conical, apical sensorial papillae. Praelabium (Fig. 27) heart-shaped and distinctly elongated, with 1 relatively long *prms*; ligula with sinuate margin and 3 piliform micro *ligs*, unequal in length; premental sclerite well visible. Labial palpi with two palpomeres; length ratio of the basal and distal palpomeres: 1:0.6; distal palpomere with one one sensillum and short, apical sensorial papillae; basal palpomere with 1 ventral sensillum, and pigmented in the basal part, and the connection with the premental sclerite seems as next palpomere. Postlabium (Fig. 27) with 3 *pms*, *pms1* located anteriorly, remaining two pairs laterally; long, almost of equal length, *pms3* distinctly shorter than *pms1* and *pms2*; surface of postlabium densely covered by distinct asperities.

*Thorax*. Prothorax distinctly smaller than meso- and metathorax. Metathorax almost of equal length as abdominal segments I–IV. Spiracle bicameral. Prothorax (Fig. 29) with 10 *prns* unequal in length, of which 8 on distinctly pigmented dorsal sclerite that is subdivided medially into two triangular plates, next two *prns* placed below; 2 long *ps* located on pigmented sclerite, and 1 *eus*. Mesothorax (Fig. 29) with 1 long *prs*; 4 long to very long *pds*, *pds2* distinctly shorter than the remaining three setae (both on weakly pigmented sclerites); 1 very short *as*; 2 short *ss*; 1 *eps*; 1 *ps* (*eps* and *ps* on weakly pigmented sclerites) and 1 *eus*. Chaetotaxy of metathorax (Fig. 29) identical to that of mesothorax. Each pedal area of the thoracic segments well separated and pigmented, with 7 long *pda*, of which 4–6 on distinctly pigmented area, unequal in length.

**Figure 28.**
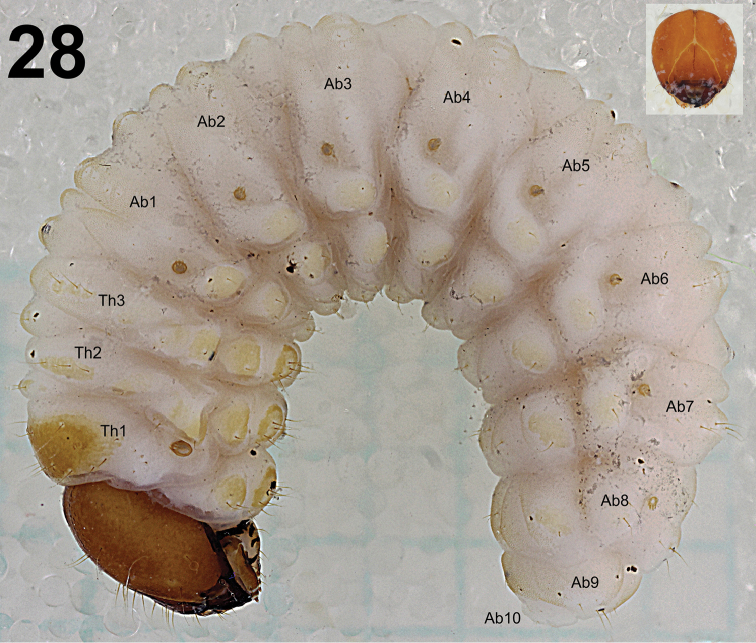
*Lixus neglectus* mature larva habitus, lateral view. Scale bar: 3 mm.

**Figures 29–31.**
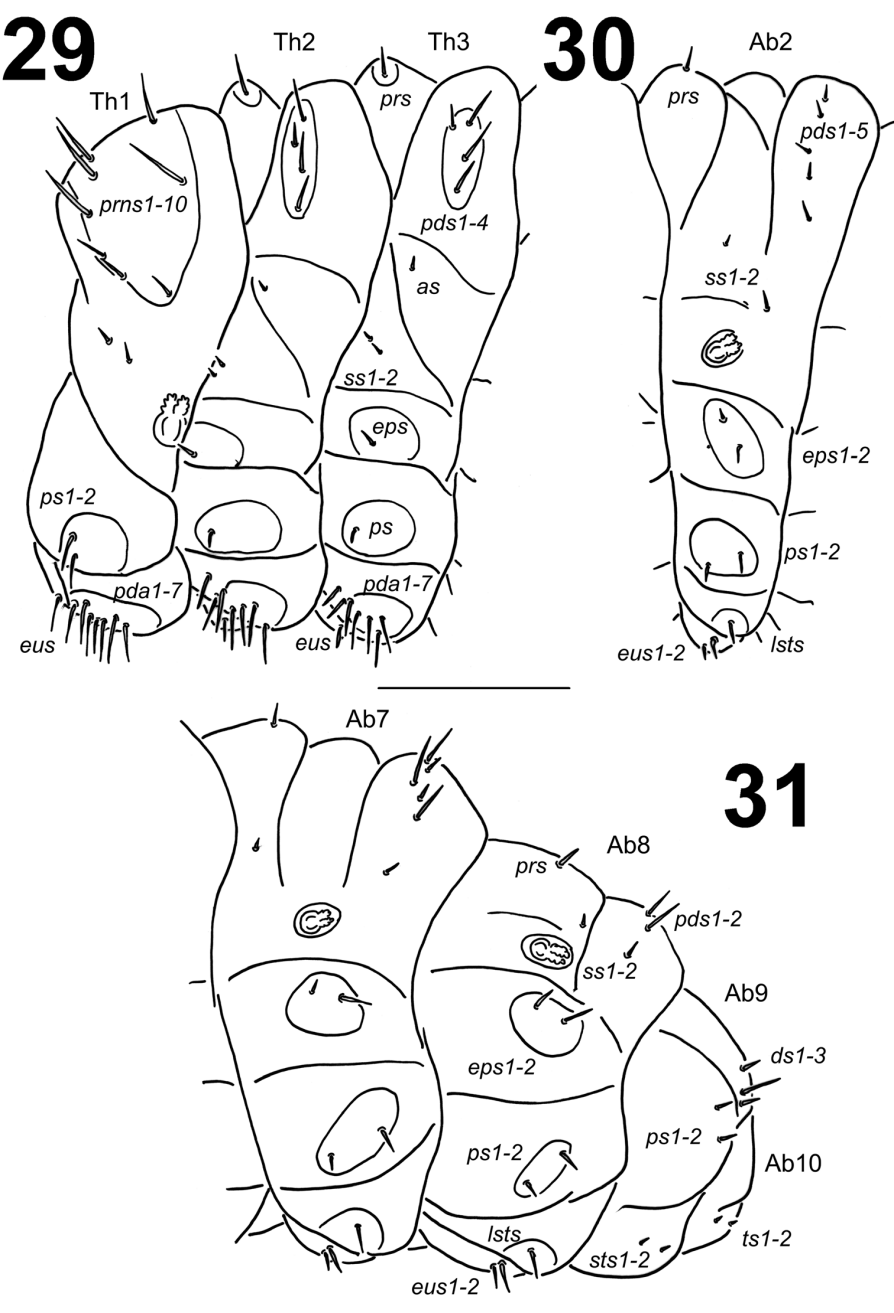
*Lixus neglectus* mature larva habitus. **29** Lateral view of thoracic segments **30** Lateral view of abdominal segment II. **31** Lateral view of abdominal segments VII–X. Scale bar: 1 mm.

*Abdomen*. Abdominal segments I–V of almost equal length and subsequent abdominal segments decreasing gradually to the terminal parts of the body. Abdominal segment X reduced to four anal lobes of unequal size, the dorsal being distinctly the largest, the lateral pair equal in size, and the ventral lobe very small. Anus located terminally. Spiracles bicameral, the eight abdominal spiracles located laterally, close to the anterior margin of abdominal segments I–VIII. Abdominal segments I–VII (Figs 30–31) with 1 *prs*; 5 relatively short *pds*, *pds2* and *pds4* on abdominal segment VII less than half of length of the three remaining setae which are twice as long as *pds* on the previous six abdominal segments; 2 *ss* of unequal length, *ss1* very short, *ss2* as long as *pds5*; 2 *eps* of unequal length; 2 relatively short *ps* of equal length; 1 long *lsts* (*eps*, *ps* and *lsts* on weakly pigmented sclerites) and 2 relatively long *eus*. Abdominal segment VIII (Fig. 31) with 1 relatively long *prs*; 2 long to very long *pds*, *pds1–2* and *pds4* lacking; 2 *ss* of unequal length, *ss1* very short, *ss2* as long as *prs*; 2 *eps* of unequal length, *eps1* relatively long, *eps2* long to very long; 2 relatively short *ps* of equal length; 1 relatively long *lsts* (*eps*, *ps* and *lsts* on weakly pigmented sclerites) and 2 relatively long *eus*. Abdominal segment IX (Fig. 31) with 3 *ds* (*ds1–2* long, *ds3* short); 2 very short *ps* and 2 very short to micro *sts*. Abdominal segment X (Fig. 31) with 2 microsetae (*ts*), on each lateral lobe.

#### Description of pupa.

*Measurements* (in mm). Body length: 9.4–12.7 (♂ 9.4–12.7; ♀ 10.0) and the widest part of the body, commonly between the apex of the meso- or metafemora: 2.8–3.8.

*Colouration*. Body yellow (Fig. 39).

**Figures 32–34.**
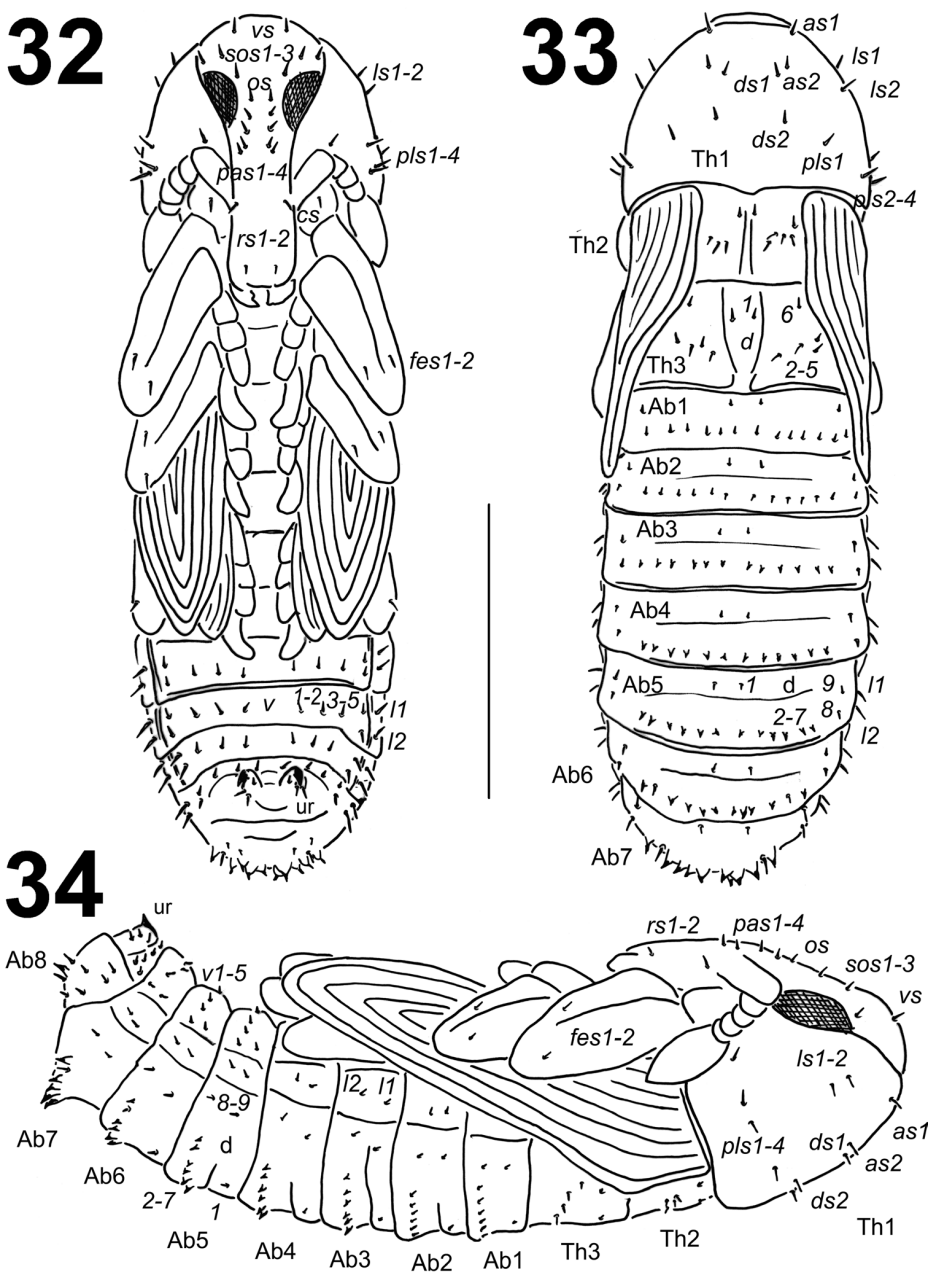
*Lixus neglectus* pupa habitus. **32** Ventral view **33** Dorsal view **34** Lateral view. Scale bar: 3 mm.

*Morphology* (Figs 32–34, 39). Body stocky, elongated, white or yellowish. Cuticle smooth. Rostrum relatively long, approximately 3.1 to 3.5 times as long as wide and extending beyond the mesocoxae. Antennae relatively long and stout. Pronotum from 1.2 to 1.3 times as wide as long. Mesonotum and metanotum of almost equal length. Abdominal segments I–V of almost equal length; abdominal segment VI semicircular and subsequent abdominal segments diminish gradually to the end of the body. Abdominal segments VII–IX distinctly smaller than other abdominal segments. Gonotheca (abdominal segment IX) in females (1 specimen) bilobed.

**Figures 35–42.**
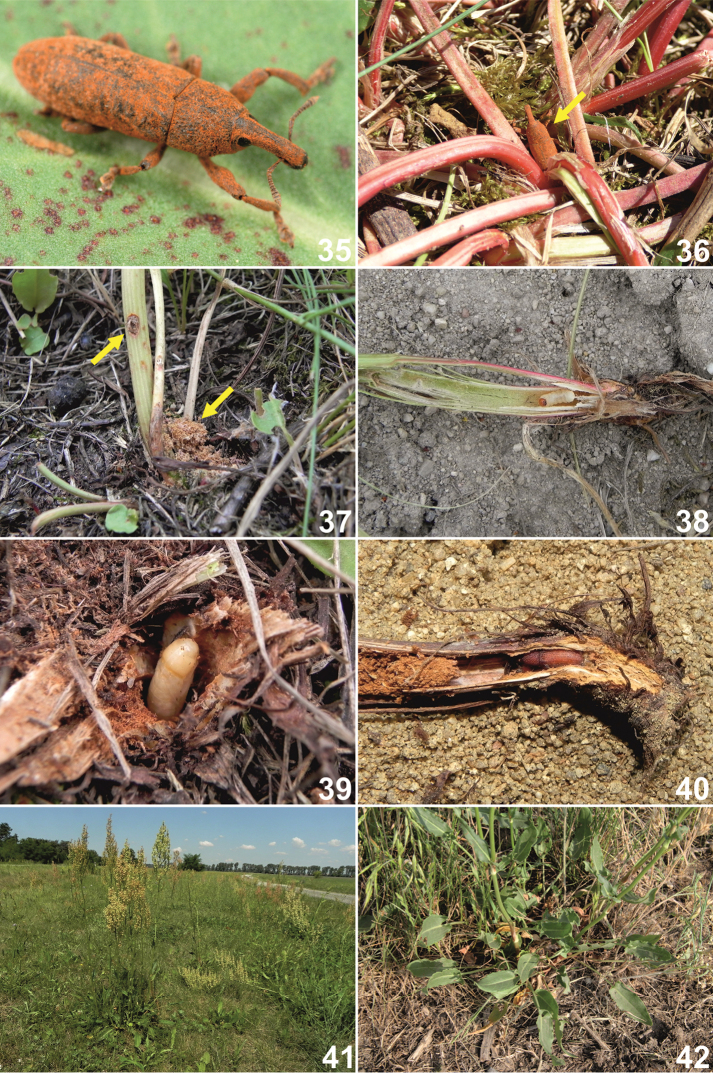
Habitats, adults, immature stages, host plants and life cycle of *Lixus neglectus*. **35** Adult **36** Adult hiding in host plant rosette **37** Ovipositional mark and larva feeding marks (frass) **38** Mature larva **39** Pupa **40** Fresh, not fully coloured adult **41** Habitat in Czech Republic with host plant, *Rumex thyrsiflorus* **42** Detail of host plant rosette.

*Chaetotaxy* (Figs 32–34). Setae relatively short, unequal in length, light yellow or orange, some setae on abdominal segments III–VIII distinctly stronger and located on protuberances. Setae well visible. Head capsule includes 1 *vs*, 3 *sos*, 1 *os* and 4 *pas*. Rostrum with 2 *rs*, *rs1* placed below antenna, *rs2* on the anterior margin. Setae on head capsule and rostrum straight, both *rs* and all *pas* distinctly shorter than the remaining setae on head, thoracic and abdominal segments. Pronotum with 2 *as*, 2 *ds*, 2 *ls* and 4 *pls*, and 1 short seta on ventral side (probably *pls5*). Dorsal parts of mesothorax with 1 seta located posteromedially, 1 seta located posterolaterally and 4 setae located along the anterior margin. Chaetotaxy of metathorax identical to that of mesothorax. Coxa with 1 very short *cs*. Each femoral apex with 2 *fes*. Dorsal parts of abdominal segments I–VIII each with 2 pairs of setae located posteriorly (*d1*, *d9*) and 7 pairs (*d2–8*) located along the anterior margins. Setae *d2–7* (on abdominal segments III–V) and setae *d2–5* and *d7* (on abdominal segments VI–VII) short, thorn-like, located on protuberances. Protuberances on abdominal segment VII distinctly prolongated. Remaining setae relatively short, hair-like. Abdominal segments I–VII with groups of 2 lateral setae and 5 pairs of ventral setae. Dorsal part of abdominal segment VIII with 1 seta located posteriorly (*d9*) and 5 pairs (*d3–7*) located along its anterior margin; *d7* thorn-like, located on protuberances; remaining setae short. Abdominal segment VIII with groups of 2 lateral setae and 5 short ventral setae. Abdominal segment IX with 2 pairs of ventral microsetae and 1 pair of short, thin setae. Urogomphi distinctly elongated, hooked, triangular.

##### Biology and ecology

*Habitats*. Adults live in dry grasslands and meadows with sandy substrates (wind-blown river sand) (Fig. 41).The meadows are often managed for hay production. Numerous specimens were also found on grassy embankments along roads.

*Adult behaviour*. Adult beetles (Fig. 35) exhibit diurnal as well as nocturnal activity. During sunny days, they spend almost all of their time hiding among the leaves of the host plant near the ground (Figs 36, 42) and rarely climb to the higher parts of the plant.

*Host plants*. Adults and their immature stages were observed exclusively on dock, *Rumex thyrsiflorus* Fingerh. (Polygonaceae) (Figs 41–42), in all of our localities. In the past, this species was only recorded from garden sorrel (*Rumex acetosa*) L. (e.g., Fremuth 1983).

*Life cycle*. *Lixus neglectus* is an univoltine species. Adults feed on leaves, and larvae are stem and root borers (Fig. 38). Females of *Lixus neglectus* bite the lower part of the stem and lay one egg in the hole (Fig. 37). Larvae feeding in the root produce orange feeding frass (Fig. 37), which is thrown out of the host plant, and its presence is a reliable indication that the plant root is occupied by a larva. One plant is most likely occupied by only one larva. Mature larvae were found from July to August. Pupation takes place in the root neck (Fig. 39), and freshly hatched individuals can be found (inside plants) beginning in the middle of August (Fig. 40). Adults do not hibernate in the host plants, so hibernation most likely occurs in the leaf litter, among dry plant debris or in the topsoil.

*Rearing of the larvae*. For laboratory breeding, 15 mature larvae were collected on July 13^th^, 2014, but only two of them pupated in our laboratory conditions. The remaining larvae died primarily due to drying of the host plants. Both of the adults hatched on July 30^th^.

## Discussion

**Comparison with larvae of other *Lixus* species.** To date, larvae of 21 *Lixus* and two *Hypolixus* species have been described (Scherf 1964; Lee and Morimoto 1988; May 1994; Nikulina 2001, 2007; Zotov 2009a, b; Nikulina and Gültekin 2011; Gosik and Wanat 2014; Skuhrovec and Volovnik 2015), but a detailed description of the pupae is known for only 8 *Lixus* species (Scherf 1964; May 1994; Zotov 2009a, b; Gosik and Wanat 2014; Skuhrovec and Volovnik 2015).

The precise general description of the larvae of the genus *Lixus*, which can be summarized by 19 character sets, has been presented by May (1994) and more in detail by Nikulina (2001) (for details, see Nikulina 2001 and Gosik and Wanat 2014). The larvae of *Lixus bituberculatus* and *Lixus neglectus* possess all of these characters, with only a few exceptions that partly result from differences in terminology (for details, see Gosik and Wanat 2014). Nikulina (2001) also published the only comprehensive and known larval key for this genus. The larva of *Lixus bituberculatus* has the closest affinity to the larva of *Lixus kiritshenkoi* Ter-Minasian, 1985 (abdominal segment X with two setae on the ventral side, lateral sides without setae, and dorsal side with less than three setae, see Table 1). The main differences are as follows (see Table 1): prothoracic tergite with 10 *prn* (vs. 13 *prn*); maxilla stipes with short 1 *mbs* (vs. without *mbs*, which could also be easily overlooked because it is very often minute); prodorsum on abdominal segment IX with only 4 *ds* (vs. 3 *ds*), and 3 short *ps* (vs. 1 *ps*). The larva of *Lixus bituberculatus* is the first of the immature stages described for the subgenus *Ortholixus*, but the larva of *Lixus neglectus* does not fit the description of any larva in the key (Nikulina 2001) because the first step provides no option for abdominal segment X to be without setae on the dorsal side (see Fig. 31). An interesting characteristic of *Lixus neglectus* is the presence of more pigmented sclerites on the larval body (see Figs 28–31, Table 1), which is similar to the description of *Lixus filiformis* (Nikulina and Gültekin 2011). In the subgenus *Dilixellus*, to which *Lixus neglectus* belongs, the larvae of four species have already been described: *Lixus bardanae* (Fabricius, 1787) (in Scherf 1964); *Lixus desbrochersi* Hoffmann, 1957 (in Lee and Morimoto 1988 as *Lixus impressiventris* Desbrochers des Loges, 1904); *Lixus probus* Faust, 1887 (in Nikulina 2001); and *Lixus punctiventris* Boheman, 1835 (in Gosik and Wanat 2014) (see Table 1). The creation of a precise key and a detailed subgeneric study of the genus *Lixus* is currently limited due to the lack of knowledge of the immature stages (see Table 1). The main problem is that we are unable to divide with certainty the morphological characteristics of this group into (i) characteristics that are useful for phylogenetics and (ii) characteristics that are useful only for species identification. Once this categorization is complete, it will be possible to apply it for future cladistics analysis, which are planned for the near future. All these morphological data should be compared and correlated with known biological data of the host plant families for the different groups of *Lixus* and also related genera, because some subgenera of *Lixus* are probably composed of different probably unrelated groups developing on quite different families of plants (Skuhrovec, Gosik, Stejskal, Trnka, Volovnik, Gültekin, unpublished data).

**Table 1.** Differential diagnosis of mature larvae of both described species and the most similar or relative species.

	Lixus (Dilixellus) bardanae (Fabricius, 1787)	Lixus (Dilixellus) desbrochersi Hoffmann, 1957	Lixus (Dilixellus) neglectus Fremuth, 1983	Lixus (Dilixellus) probus Faust, 1886	Lixus (Dilixellus) punctiventris Boheman, 1835	Lixus (Epimeces) filiformis (Fabricius, 1781)	Lixus (Eulixus) kiritshenkoi Ter-Minasian, 1985	Lixus (Ortholixus) bituberculatus Smreczyński, 1968
Endocarina	unknown	present, length unknown	more than the half length of frons	short	more than the half length of frons	more than the half length of frons	more than the half length of frons	more than the half length of frons
Number of *des*	unknown	4	5	5	5	5	5	5
Number of *fs*	unknown	5	5	3	5	5	5	5
Number of *les*	unknown	2	2	unknown	3	1	unknown	2
Number of *ves*	unknown	2	2	unknown	2	unknown	unknown	2
Position of *lrms1-3*	in a triangle	only 2 setae	in a triangle	in a triangle	in a triangle	in a triangle	in a line	in a triangle
Number of *als*	unknown	6	4	4	5	3	3 (or 4)	4
Number of *ligs*	2	2	3	3	2	2	1	3
Number of *mbs*	1	unknown	1	1	2 ?	1	0	1
More pigmented sclerites on larval body, not only on pronotum	unknown	absent	present	absent	absent	present	absent	absent
Number of *prns*	unknown	8	10	10	10	10	13	10
Number of *pds* on Abd. seg I- VII	unknown	5	5	unknown	6	6	unknown	6
Number of *ds* on Abd. seg IX	unknown	unknown	3	unknown	6	2	3	4
Number of *ps* on Abd. seg IX	unknown	unknown	2	unknown	3	2	1	3
Number of *ts* on dorsal lobe on Abd. Seg. X	unknown	unknown	0	7 *	0	0	2 *	0
Number of *ts* on ventral lobe on Abd. Seg. X	unknown	unknown	0	2 *	0	0	2 *	0
Number of *ts* on lateral lobe on Abd. Seg. X	unknown	unknown	2	0 *	3	3 (dorsoventral)	0 *	3

* Nikulina (2001) listed number of *ts* on sides, not on lobes.

May (1993) considered the increased number of *pds* on the meso- and metathorax and abdominal segments I–VII and the increased number of setae on the epipharyngeal lining (*als*) (i.e., higher than the most frequent number of setae in weevils) as diagnostic of the mature larva of the Lixinae subfamily, and the descriptions of mature larvae from the tribe Lixini (*Larinus* species: Zotov 2009a, 2010; Gosik and Skuhrovec 2011; *Lixus* species: Scherf 1964; Lee and Morimoto 1988; May 1994; Nikulina 2001, 2007; Zotov 2009a, b; Nikulina and Gültekin 2011; Gosik and Wanat 2014; Skuhrovec and Volovnik 2015; *Rhinocyllus conicus*: May 1994) fit this diagnosis, as do all known species from the tribe Cleonini (Stejskal et al. 2014, Trnka et al. 2015). Currently, the comparison of both tribes, including key and detailed generic studies, is impossible due to our limited knowledge of the immature stages. A categorization of the morphological characteristics of Cleonini and a comparison of both tribes is planned following a detailed study of the genus *Lixus* (see the previous paragraph). The presence of 5 *vms* in the maxillary mala observed in all Lixinae and also most curculionids could be helpful as differential character from the root feeder larvae in Entiminae which have only 4 *vms* (Marvaldi 1998a, b).

## Biology and ecology

The biology and development of these two *Lixus* species are very similar as both species are stem and root (crown) borers. In the genus *Lixus*, root borers, such as Lixus (Ortholixus) angustus (Herbst, 1795); Lixus (Compsolixus) ochraceus Boheman, 1842; Lixus (Dilixellus) punctirostris Boheman, 1842; Lixus (Dilixellus) punctiventris Boheman, 1835; and Lixus (Ortholixus) vilis (Rossi, 1790) (Dieckmann 1983) have not been identified as frequent. The prevailing nocturnal activity of the adults and the species hidden life habits are probably the main reasons why these species have not been found elsewhere, and it is very difficult to confirm them at a locality. The majority of the Central European Lixinae species require a specific habitat disturbance regime that results in sparse vegetation cover (Stejskal and Trnka 2013, 2014). In both species, it seems that adults and the immature stages prefer places with pasture vegetation or meadows that are managed for hay production.

This is the first report of *Lixus bituberculatus* from Romania, and it probably has a larger area of distribution including Bulgaria, Hungary, Romania and Slovakia (Gültekin and Fremuth 2013; Stejskal and Trnka 2014). Based on our observations *Lixus bituberculatus* appears to be oligophagous on Asteraceae; all of our specimens were only found on *Cichorium intybus*, which originates from the Mediterranean but has been introduced to North America, southern Africa and New Zealand (Dvořáková 2005). It seems that this weevil is not specialized on only one plant species because it was found on *Crepis* sp. as well as *Picris* sp. in Slovakia, so its suitability as a candidate for the biological control of *Cichorium intybus* is questionable. Only a host plant choice test can help us to determine all of the host plant species and the preferences of this species.

*Lixus neglectus* has been found exclusively on *Rumex thyrsiflorus*, but the distribution of its host plant covers the majority of Europe (Kubát 1990). However, this beetle is only known from a very small and specific area. The sorrel *Rumex thyrsiflorus* is considered to be a naturalized neophyte in Central Europe (Danihelka et al. 2012), but its origin remains unknown (Kubát 1990). This *Lixus* species seems to be monophagous, and its only host plant, *Rumex thyrsiflorus*, is not considered to be harmful and has not yet been introduced to other continents or countries. Therefore, it is not necessary to utilize this weevil to regulate its host plant despite its suitability as a biological control agent, but this must be validated by future studies. The distribution of *Lixus neglectus* has to be confirmed, especially across the wider area of distribution of *Rumex thyrsiflorus*. This weevil can be easily overlooked due to its cryptic way of life, but the easiest way to confirm its presence in the field is to search for frass at the root crown of the host plant. This frass is a very unique behaviour within the genus *Lixus*. To date, this weevil has only been recorded from *Rumex acetosa* (e.g., Fremuth 1983; Koch 1992; Böhme 2001), which is likely due to misidentification as occurred in our case (Trnka and Stejskal 2014). It is possible that *Lixus neglectus* historically lived on *Rumex acetosa* but has recently come to occupy a new available ecological niche on expanding *Rumex thyrsiflorus* (from ca 15th century). However, because each of the host plants prefer different habitats, this explanation is quite unlikely. The common sorrel, *Rumex acetosa*, prefers wet habitats, whereas the compact dock, *Rumex thyrsiflorus*, prefers dry ones (Kubát 1990). Both sorrels are also known as host plants for some other weevils, but there does not seem to be any competition among the species. Development on *Rumex acetosa* is also known in one other *Lixus* species, *Lixus bardanae* (Fabricius, 1787), but this weevil is a typical stem borer. Thus there is no competition between it and *Lixus neglectus* (Dieckmann 1983). A similar situation occurs in the development of some Apionids (e.g., *Apion cruentatum* Walton, 1844 or *Perapion oblongum* (Gyllenhal, 1839)), whose larvae also feed only in the stems (Dieckmann 1977). Furthermore, both plants host the oligophagous weevil species *Marmaropus besseri* Gyllenhal, 1837 (Dieckmann 1972) in their stems and roots, and this species has been recently expanding along with *Rumex thyrsiflorus* (Rheinheimer and Hassler 2010). Both of these weevils belong to the same guild (stem and root (crown) borers), so there could be some competition. However, this requires more information on the timing of their development as well as some of the other abiotic and/or biotic effects inside the plant. Thus, the complete switch in host plant by *Lixus neglectus* seems unlikely, mainly due to its rarity despite the recent expansion of its host plant. It is more probable that its preference for *Rumex thyrsiflorus* as a host plant is recent, but this could only be resolved through a host plant choice test.

Knowledge of the immature stages and life histories of insects can help protect endangered species (including the species presented here) more effectively. The detailed descriptions of the larva and pupa and their comparison with known descriptions reported here demonstrates the possibility of identifying species in their immature stages. Future detailed biological and morphological studies can yield unique information on the factors determining host specificity in this insect group and will provide useful background information for planning efficient biocontrol of invasive plant species. The issue of using some insects as biological control agents is a key topic in both the basic and applied research on invasive plants. Our results will significantly contribute to basic research but will also have practical implications for conservation biology and/or biological control.

## Supplementary Material

XML Treatment for Lixus (Ortholixus) bituberculatus

XML Treatment for Lixus (Dilixellus) neglectus
